# Aspirin Inhibits Fibronectin Expression and Reverses Fibronectin-Mediated Cell Invasiveness by Activating Akt Signaling in Preeclampsia

**DOI:** 10.3390/ph15121523

**Published:** 2022-12-08

**Authors:** Mei-Tsz Su, Ching-Wei Tsai, Pei-Yin Tsai, Chia-Yih Wang, Hui-Ling Tsai

**Affiliations:** 1 Department of Obstetrics and Gynecology, National Cheng Kung University Hospital, College of Medicine, National Cheng Kung University, 138 Sheng-Li Road, Tainan 70101, Taiwan; 2 Department of Obstetrics and Gynecology, Tainan Hospital, Ministry of Health and Welfare, Tainan 700007, Taiwan; 3 Department of Internal Medicine, Tainan Hospital, Ministry of Health and Welfare, Tainan 700007, Taiwan; 4 Department of Cell Biology and Anatomy, College of Medicine, National Cheng Kung University, Tainan 70101, Taiwan; 5 Institute of Basic Medical Sciences, College of Medicine, National Cheng Kung University, Tainan 70101, Taiwan

**Keywords:** aspirin, fibronectin, preeclampsia, trophoblast invasion, Akt

## Abstract

Preeclampsia is a severe gestational hypertensive disorder that may lead to maternal multiple organ dysfunction and adverse fetal outcomes. Aspirin provides a protective effect by reducing the risk of preeclampsia; however, its mechanism of action is unclear. Fibronectin (FN) is a key factor in cell motility and is associated with preeclampsia. Here, we demonstrated that cellular FN expression was elevated in the placenta of preeclamptic patients. The functional roles of plasma and cellular FN in trophoblasts were investigated by treating HTR-8/SVneo cells with exogenous recombinant human FN protein (rhFN) and siRNA, respectively. Trophoblast migration and invasion were inhibited by rhFN and facilitated by FN knockdown. Moreover, rhFN activated ERK and Akt signaling in trophoblasts, and FN-suppressed cell motility was rescued by ERK and/or Akt inhibitors. In this study, aspirin suppressed trophoblast cellular FN expression and reversed FN-mediated cell functions, including cell migration, invasion, and ERK/Akt signal changes. Taken together, the results of this study revealed the effects of FN on trophoblast motility and signaling; aspirin inhibits FN expression and reverses FN-mediated trophoblast biology. These results provide a drug mechanism for disease prevention and a target for preeclampsia intervention.

## 1. Introduction

Preeclampsia is one of the most common complications of pregnancy, with an incidence of 2–8% worldwide [1]. The clinical presentation of preeclampsia includes new-onset hypertension; proteinuria; and multiple organ dysfunction, such as eclampsia, pulmonary edema, and renal insufficiency, and may cause maternal or fetal mortality in some severe cases [1,2]. Several mechanisms have been proposed for the pathogenesis of preeclampsia, such as placental dysfunction, vascular endothelial dysregulation, and maladaptation of uterine spiral arterial remodeling. Insufficient trophoblast invasion and uterine artery remodeling during the first trimester result in abnormal perfusion of uterine blood flow from the maternal circulation to the fetus and vascular dysfunction with abnormal angiogenic factor secretion in later gestation, which triggers the development of preeclampsia [3].

Fibronectin (FN) is a large glycoprotein involved in cell migration, adhesion, and tissue repair [4]. FN is widely synthesized in several cell types and exists in two forms, plasma and cellular FN. Both isoforms are generated from a single gene by alternative splicing. [5]. Plasma FN is mainly synthesized in the liver and released into circulation in a soluble but inactive form unless bound to its receptor on the cell surface to activate downstream signaling. Cellular FN is produced by numerous cell types in an insoluble form as a part of the extracellular matrix (ECM) and provides a scaffold for cells to alter the composition of ECM during development and tissue remodeling/repair. [6]. In the early human placenta, FN is expressed on infiltrating extravillous trophoblasts and endovascular trophoblasts, and it is believed to regulate trophoblast adhesion and migration into maternal tissue [7]. Previous studies have indicated an association between higher FN expression in the placenta and/or plasma of preeclamptic patients, and some have speculated that this may be derived from vascular injury release, increased FN production, or enzyme degradation; however, the exact etiology is not clear [8,9,10,11]

Aspirin has recently been used as an effective agent to decrease the risk of preeclampsia in high-risk pregnant women [12,13]. The mechanism underlying the protective effect of aspirin has not yet been fully elucidated. Aspirin has been clinically used for decades for the primary and secondary prevention of cardiovascular disease, benefiting from antiplatelet and antithrombotic effects through cyclooxygenase (COX) inhibition and related suppression of prostaglandin production [14,15]. Furthermore, aspirin was repurposed for its antitumor effects in several cancers through different mechanisms, including anti-inflammatory tumorigenesis, immune regulation in the tumor microenvironment, and regulation of prostaglandin metabolism [16,17]. Aspirin has been shown to alleviate hypoxia-induced trophoblast apoptosis and invasion [18] and enhance the function of uterine mesenchymal stem cells [19]. Although the effects of aspirin on preeclampsia prevention have been studied, the role of placental FN in pregnancy and the mechanism of its involvement in preeclampsia have not been explored. Here, we investigated the role of FN in trophoblasts and the effects of aspirin on FN-mediated cell biology and signaling. This study provides a possible mechanism for aspirin efficacy in preeclampsia prevention.

## 2. Results

### 2.1. Fibronectin Protein Expression Was Elevated in the Placentas of Preeclamptic Patients

**Figure 1:**
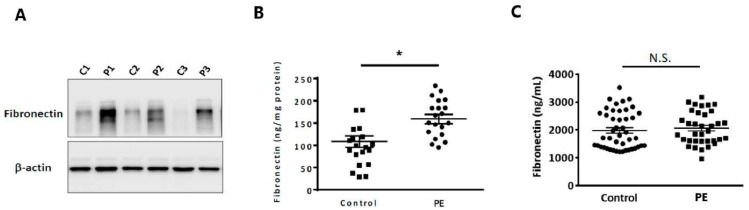
Placental fibronectin (FN) expression is elevated in patients with preeclampsia. The protein concentration of FN in the placenta was measured in preeclamptic patients (P1-3 and PE) and women with normal pregnancies (C1, C1, C3, and control) using (**A**) Western blotting (n = 3 in both groups) and (**B**) ELISA (n = 20 in both groups). (**C**) The plasma FN concentration was measured in preeclamptic patients (n = 35) and normal controls (n = 45) using ELISA. Data are presented as the means ± SEMs. N.S. denotes no significance, * *p* < 0.05 compared with the corresponding control.

The expression level of placental cellular FN was elevated in preeclamptic patients compared with healthy controls, as determined by Western blotting (Figure 1A) and ELISA (n = 20 in both groups) (Figure 1B), whereas the circulating plasma FN concentration did not show a significant difference (n = 35 and n = 45, respectively) using ELISA (Figure 1C).

### 2.2. Fibronectin Inhibits Trophoblast Invasion and Migration without Affecting Cell Viability

**Figure 2:**
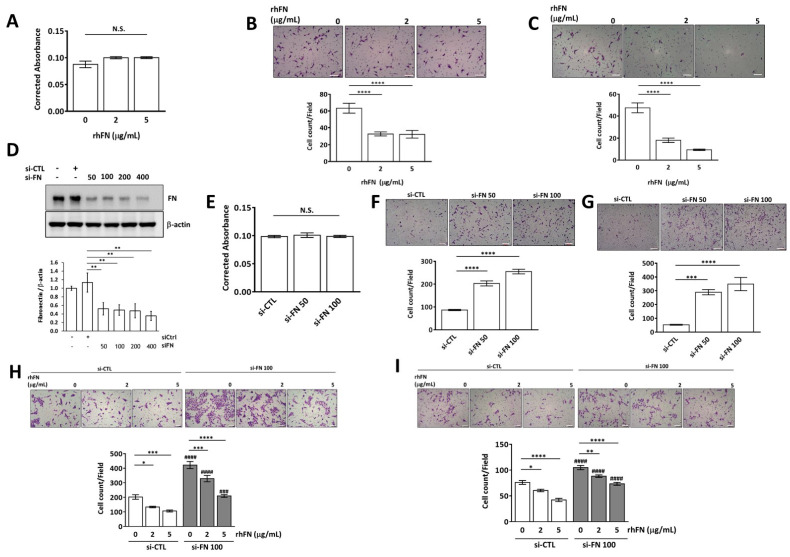
Fibronectin (FN) inhibits trophoblast migration and invasion without affecting cell viability. HTR-8/SVneo cells were treated with fibronectin (0, 2, and 5 μg/mL, rhFN) in serum-free medium for 24 h and then processed for (**A**) cell viability measurements after 24 h of culture and (**B**,**C**) cell migration and invasion assays for the next 24 h using a transwell system. HTR-8/SVneo cells were transfected with scrambled siRNA (si-CTL) or si-FN for 24 h, and cells were subjected to (**D**) cellular FN expression in the cell lysate using Western blotting and quantification of Western blotting, (**E**) cell viability, (**F**) cell migration, and (**G**) cell invasion assays. After FN knockdown in HTR-8/SVneo cells with si-FN for 24 h, cells were treated with rhFN (5 μg/mL) for another 24 h before (**H**) migration and (**I**) invasion assays. Data are presented as the mean ± SEMs. N.S. denotes no significance, * *p* < 0.05, ** *p* < 0.01, *** *p* < 0.005, **** *p* < 0.001 compared with the corresponding control. ^###^ *p* < 0.005, ^####^ *p* < 0.001 compared with samples at the same rhFN concentration. Scale bar: 100 μM.

HTR-8/SVneo cells were treated with 2 and 5 μg/mL rhFN for 24 h and then subjected to cell viability, migration, and invasion assays. Although cell viability was not affected, cell invasion and migration abilities were suppressed after rhFN treatment (Figure 2A–C). We then explored functional alterations in trophoblasts following cellular FN knockdown. After transfecting HTR-8/SVneo cells with either si-CTL or si-FN (50 and 100 nM) for 24 h, trophoblast viability, migration, and invasion assays were performed (Figure 2D–G). FN-knockdown trophoblasts were shown to have enhanced cell migration and invasion abilities without affecting cell viability. In subsequent experiments, we evaluated the effect of rhFN on FN-knockdown trophoblasts. HTR-8/SVneo cells were transfected with si-CTL or si-FN (100 nM) for 24 h and subsequently treated with rhFN (0, 2, and 5 μg/mL) for another 24 h. The cells were used for motility studies in a transwell system. Although rhFN suppressed cell migration and invasiveness, FN-knockdown trophoblasts had better cell motility than those without FN knockdown (Figure 2H,I).

### 2.3. Fibronectin Regulates Trophoblast Invasiveness and Migration by Activating the ERK and Akt Signaling Pathways

**Figure 3:**
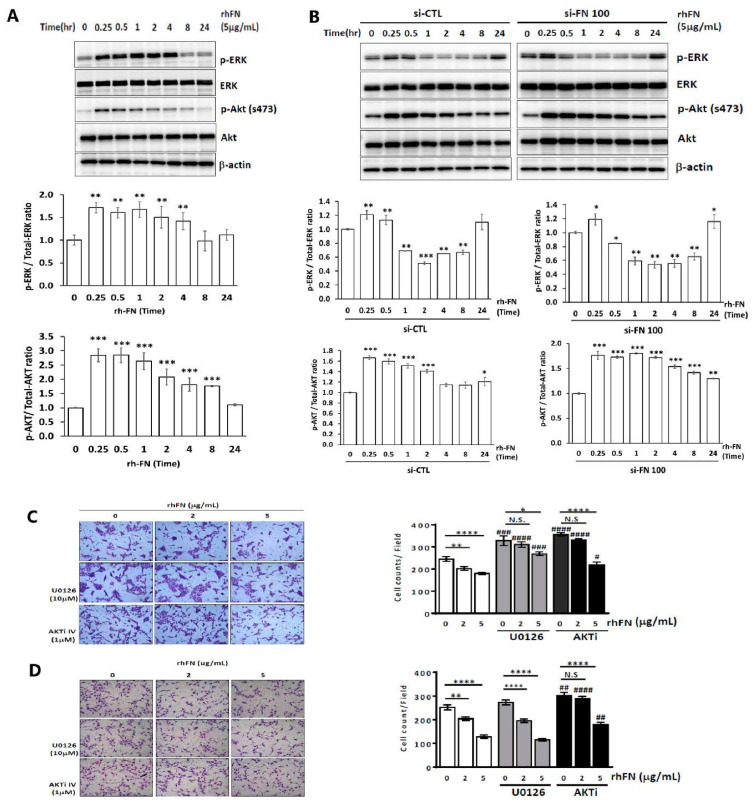
Fibronectin (FN) regulates trophoblast invasiveness and migration by activating ERK and Akt signaling. Phospho-ERK, ERK, phospho-Akt, and Akt were analyzed at different timepoints (0, 0.25, 0.5, 1, 2, 4, 8, and 24 h) after cells were treated with rhFN (5 μg/mL) using Western blotting and quantification of Western blotting in (**A**) HTR-8/SV neo and (**B**) FN-knockdown cells. (**C**,**D**) HTR-8/SVnel cells were pretreated with rhFN (5 μg/mL) for 24 h and then U0126 (10 μM) or Akt inhibitor (1 μM) for another 4 h before (**C**) cell migration and (**D**) cell invasion assays. Data are presented as the means ± SEMs. N.S. denotes no significance, * *p* < 0.05, ** *p* < 0.01, *** *p* < 0.005, **** *p* < 0.001 compared with the control treated with the same inhibitor. ^#^ *p* < 0.05, ^##^ *p* < 0.01, ^###^ *p* < 0.005, ^####^ *p* < 0.001 compared with samples at the same rhFN concentration without ERK treatment or Akt inhibitors. Scale bar: 100 μM.

After treating HTR-8/SVneo or FN-knockdown cells with rhFN (5 μg/mL) in 0% FBS, signal proteins were observed at different time points (0, 0.25, 0.5, 1, 2, 4, 8, and 24 h) by collecting the cell lysates using Western blotting. Signals for phospho-ERK and phospho-Akt (S473) were activated and lasted for at least 4 h in HTR-8/SVneo cells (Figure 3A). For FN-knockdown HTR-8/SVneo cells (si-FN 100 nM), phospho-ERK and phospho-Akt (S473) signals were also activated after rhFN treatment (Figure 3B). We then added rhFN-pretreated trophoblasts with ERK/Akt inhibitors (10 μM U0126 and 1 μM AktI) for 4 h to evaluate cell motility. The inhibitory effects of rhFN on cell migration were partially rescued by the treatment of trophoblasts with ERK and AKT inhibitors, whereas cell invasiveness was rescued by the Akt inhibitor only (Figure 3C,D).

### 2.4. Aspirin Inhibits Cellular Fibronectin Expression and Reverses Fibronectin-Mediated Cell Motility in Trophoblasts through the ERK and Akt Pathways

**Figure 4:**
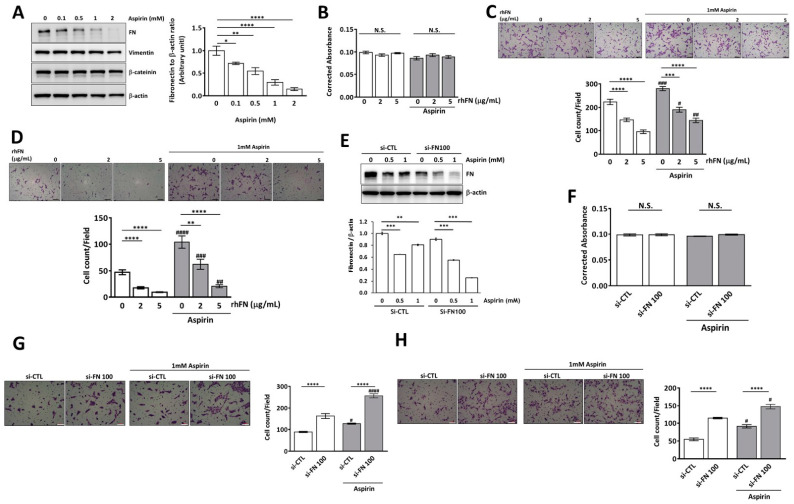
Aspirin inhibits fibronectin (FN) expression and reverses FN-mediated trophoblast migration and invasion in HTR-8/SVneo cells. (**A**) Different doses of aspirin (0, 0.1, 0.5, 1, and 2 mM) were used to treat HTR-8/SVneo cells for 24 h, and then cellular FN expression in the cell lysate was analyzed and quantified using Western blotting. (**B**–**D**) HTR-8/SVneo cells were treated with rhFN (5 μg/mL) for 24 h and then aspirin (1 mM) for another 24 h, and the cells were then subjected to (**B**) cell viability, (**C**) cell migration, and (**D**) cell invasion assays. After FN knockdown of HTR-8/SVneo cells with si-FN (100 nM) for 24 h, cells were treated with aspirin (1 mM) for another 24 h before determining (**E**) cellular FN expression using Western blotting and its quantification, (**F**) cell viability, (**G**) migration, and (**H**) invasion assays. Data are presented as the means ± SEMs. N.S. denotes no significance, * *p* < 0.05, ** *p* < 0.01, *** *p* < 0.005, **** *p* < 0.001 compared with the control treated with the same reagents. ^#^ *p* < 0.05, ^##^ *p* < 0.01, ^###^ *p* < 0.005, ^####^ *p* < 0.001 compared with the samples at the same rhFN or si-FN concentration without aspirin treatment. Scale bar: 100 μM.

After treating HTR-8/SVneo cells with different concentrations of aspirin (0, 0.1, 0.5, 1, and 2 mM), the cellular FN protein expression was downregulated in a dose-dependent manner (Figure 4A). To further investigate the effect of aspirin on the motility of FN-knockdown and non-FN-knockdown trophoblasts, HTR-8/SVneo cells were treated with si-FN and rhFN for the first 24 h and then with aspirin (1 mM) for another 24 h before cell viability and motility experiments. Aspirin did not affect cell viability or enhance cell migration and invasion (Figure 4B–D). Moreover, aspirin at least partially reversed the rhFN-mediated inhibitory effect on trophoblast invasion and migration (Figure 4C,D). In FN-knockdown trophoblasts, aspirin suppressed cells with low cellular FN expression at an even lower FN concentration (Figure 4E). Aspirin promoted cell migration and invasion without affecting cell viability in FN-knockdown trophoblasts (Figure 4F–H).

## 3. Discussion

In the present study, placental cellular FN expression was upregulated in preeclampsia patients. Thus, we explored the functional roles of plasma and cellular FN in trophoblasts and evaluated the effects of aspirin on FN-mediated cell functions. Both exogenous recombinant and cellular FN inhibited trophoblast migration and invasion, and rhFN exerted this effect through the ERK and AKT pathways. Aspirin not only suppressed the expression of cellular FN but also reversed FN-mediated trophoblast motility. These results suggest that aspirin may act by alleviating or reversing the pathogenic processes that plasma and cellular FN exert on preeclampsia, and this provides a basis for the mechanism of aspirin in preeclampsia prevention.

During embryo implantation and placental development, the composition and structure of the extracellular matrix (ECM) in the placenta and feto-maternal interface are dynamic. The placenta is abundant in diverse extracellular matrices, such as fibrillar collagens, type IV collagen, and fibronectin [20]. Fibronectin is involved in the regulation of blastocyst implantation and placental organization [21,22]. The invasion and migration of trophoblasts into the decidua and myometrium are critical processes in establishing a blood and oxygen supply in normal pregnancy, and insufficient trophoblast invasion and tissue remodeling in this step may lead to preeclampsia [23]. The binding interaction between trophoblasts and the ECM is mainly mediated by integrins, and plasma FN has been demonstrated to anchor villi on collagen or facilitate trophoblast migration by binding to the transmembrane integrin α5β1 receptor and others, such as α3β1, αvβ3, and αvβ6 [22,24,25]. By alternative splicing, cellular FN can be expressed in the form of various isoforms in different cells, tissues, and developmental stages [6]. Therefore, cell invasiveness is controlled by tissue- and cell-specific differences [26]. MAPK (including ERK1/2, JNK, and p38) and PI3K-Akt signaling have been shown to regulate trophoblast migration and invasion in many studies, and some effects were stimulating [27,28], while others were inhibitory [29,30,31]. The regulation of trophoblast motility by MAPK and PI3K signaling depends on the ligands and receptors on the host cells and downstream molecules that activate MAPK and PI3K signaling, such as transcription factors, MMP2, and MMP9. In this study, rhFN and cellular FN inhibited trophoblast invasion and migration. Moreover, exogenous recombinant FN activated both ERK and AKT signaling in trophoblasts. We further determined that trophoblast migration is governed by ERK and AKT signaling, whereas cell invasiveness is governed by AKT.

In studies exploring the role of FN in preeclampsia, most researchers have investigated the association between FN and preeclampsia and indicated that FN is a useful predictor of preeclampsia development [9,10,32,33] Most related studies have shown elevated plasma and/or placental FN concentrations in preeclamptic patients, and this change could be detected as early as 16 weeks of gestation [10]. Plasma FN concentration has also been reported to be related to blood pressure and signs of organ involvement in preeclampsia [10]. Moreover, hypertension itself was demonstrated to induce alternatively spliced forms of fibronectin in rat aortas [34]. Although mechanisms have been proposed to explain the elevation of FN levels in preeclampsia, such as vascular injury release, increased FN production, and enzyme degradation, the obscure role of FN in preeclampsia remains unclear. In this study, we showed that both rhFN and cellular FN inhibited trophoblast invasion and migration, suggesting that both plasma and cellular FN may affect normal placental development. Although no difference was observed in plasma FN levels between preeclamptic patients and controls in this study, trophoblasts secreted FN protein into the extracellular space (data not shown) and may exert their effect in the placenta through an autocrine effect. Therefore, trophoblasts were immersed in higher levels of FN in the placental microenvironment of preeclamptic patients and had impaired cell motility via the upregulation of inner cellular FN and an autocrine effect of FN secreted from nearby cells.

Aspirin is an effective prophylactic agent used in early pregnancy to reduce the risk of preeclampsia development [35]. The mechanism of action of aspirin has been proposed to involve the inhibition of sFlt-1-mediated cell invasion and apoptosis, STOX1-induced cell proliferation and migration, miR-200/ZEB1-regulated epithelial-mesenchymal transition in trophoblasts, and COX-2 and related inflammatory pathways in placental function [31,36,37,38,39]. The effects of aspirin on FN expression and cell function have been demonstrated in other cell and disease models. Aspirin inhibited cell adhesion to FN and the cell invasiveness of PC-3 (a bladder cancer cell line) [40] and suppressed scar markers, including FN, and scar formation through JNK/STAT-3 in a tendon injury rat model [41]. Moreover, aspirin abolished FN-induced MMP2 upregulation in rhabdomyosarcoma cells through the COX-2 and PGE2 pathways [42]. In our experiments, aspirin suppressed the expression of cellular FN and the FN-mediated inhibitory effects on cell invasion and migration in trophoblasts. However, this study had some limitations. First, the study design for analyzing plasma and placental FN expression was retrospective, and the clinical sample size was relatively small. Maternal circulating FN at term may not reflect the insufficient trophoblast invasion and dysfunction caused by upregulated placental FN from early pregnancy. The inclusion of more patients with severe features or with early-onset preeclampsia may have resulted in differences in plasma FN levels between the two groups. Second, animal experiments could be used to delineate the exact functional role of FN in trophoblast biology of the early placenta and its impact on preeclampsia development. Nevertheless, we demonstrated that the functional role of cellular and plasma FN on first-trimester trophoblast invasion was inhibitory, and this effect was reversed by a preeclampsia prevention agent, aspirin, which is effective in preventing early-onset preeclampsia development when used before 16 weeks of gestation. Rescue of aspirin may provide a plausible mechanism for the prevention of preeclampsia.

## 4. Materials and Methods

### 4.1. Human Specimens

This study was approved by the institutional review board of the National Cheng Kung University Hospital (Tainan, Taiwan) (#A-ER-104-209), and all participants provided informed consent to participate in the study. Preeclampsia was defined according to the guidelines released by the American College of Obstetricians and Gynecologists. Normal controls were defined as healthy-term pregnant women without any systemic diseases or pregnancy complications. Maternal blood was obtained during admission before delivery, and the placenta was obtained immediately after delivery. Clinical samples and tissues were placed in microcentrifuge tubes (SSI, Lodi, CA, USA) and stored at −80 °C until analysis.

### 4.2. Cell Cultures and Treatments

The HTR-8/SVneo trophoblast cell line was a generous gift from Dr. Charles Graham (Queen’s University, Kingston, ON, Canada) and was established using human first-trimester chorionic villi (weeks 8–10). HTR-8/SVneo cells were grown in an RPMI 1640 medium (Invitrogen, Grand Island, NY, USA) supplemented with 10% fetal bovine serum (FBS) and 100 IU/mL penicillin-streptomycin. The cells were cultured in a humidified incubator with 5% CO_2_ at 37 °C.

### 4.3. Small Interfering RNA (siRNA) Transfection Experiments

Fibronectin was depleted from trophoblasts using annealed and scrambled siRNAs. HTR-8/SVneo cells were transfected with scrambled siRNA (si-CTL) or si-FN pool (50, 100, 200 nM) (Sigma-Aldrich, Saint Louis, MO) using Lipofectamine™ 2000 (Thermo Fisher Scientific, Waltham, MA) for 24 h. siRNA sequences were as follows: si-CTL sequence 5′-GAUCAUACGUGCGAUCAGA[dT][dT]-3′, si-FN sequence (1) 5′-CTGAAGAGACTTGCTTTGA-3′, si-FN sequence (2) 5′-CACTTATGAGCGTCCTAAA-3′; si-FN sequence (3) 5′-CAATTACACTGATTGCACT-3′, and si-FN sequence (4) 5′-GTGTGATCCCGTCGACCAA-3′. For recombinant fibronectin (FN), ERK or AKT inhibitors, and aspirin experiments, HTR-8/SVneo cells were transfected with si-FN (100 nM) using Lipofectamine 2000 for 24 h and then treated with exogenous recombinant human FN (ProSpec, Ness Ziona, Israel), inhibitors (U0126 or AKT inhibitor IV), or aspirin (acetyl salicylic acid; YungShin, Taiwan) before processing for further analysis. The experiments were performed in triplicate.

### 4.4. Cell Viability Assays

The effects of si-FN, recombinant human FN (Prospec, Ness Ziona, Israel), and/or aspirin on trophoblast viability was assessed. The cell lines were transfected with scrambled siRNA or si-FN at different concentrations (0, 50, 100, and 200 nM) and grown at 2 × 10^3^ cells/well in a 96-well plate in RPMI medium without FBS for 24 h. For the recombinant human fibronectin experiment, the cells were treated with fibronectin (0, 2, and 5 μg/mL) in serum-free media for 24 h. Cell numbers were determined using PrestoBlue^TM^ cell viability reagent (Invitrogen, Carlsbad, CA) after 1 and 2 days of cell culture. After adding 100 μL of PrestoBlue^TM^ reagent to each well for 3 h, the absorbance at 570 nm was measured using a microplate reader (FlexStation 3; Molecular Devices, Sunnyvale, CA, USA) with 600 nm as the reference wavelength. All treatments were performed in triplicate, and each experiment was performed at least three times.

### 4.5. Transwell Invasion and Migration Assays

Falcon cell culture inserts (8-μm pores; Corning, New York) were used to evaluate cell migration and invasion ability. For invasion experiments, Matrigel (1 mg/mL; BD Biosciences, San Diego, CA, USA) was used to cover the upper side of the inserts, whereas the lower chamber contained a complete growth medium. Cells were seeded in the upper chamber in serum-free media and allowed to invade the matrix or migrate through the other side of the membrane for 24 h.

To evaluate the effect of aspirin on rhFN-pretreated or FN-knockdown trophoblasts, HTR-8/SVneo cells were pretreated with rhFN (5 μg/mL) or transfected with si-FN (100 nM) for 24 h and then treated with aspirin (1 mM) in serum-free media for another 24 h. For the ERK and Akt inhibitor experiments, HTR8/SVneo cells were treated with rhFN (0, 2, and 5 μg/mL) for 24 h, followed by treatment with 10 μM U0126 (Cell Signaling, Danvers, MA, USA) or 1 μM Akt inhibitor IV (CAS 681281-88-9; Calbiochem, Tokyo, Japan) in serum-free media for another 4 h. After pretreatment, the cells were used for invasion or migration studies. The invading cells were fixed in 100% methanol, stained with Giemsa’s azur eosin methylene blue solution (Merck, Darmstadt, Germany), and quantified under a light microscope (Olympus, Solna, Sweden). The assays were performed in three independent experiments.

### 4.6. Enzyme-Linked Immunosorbent Assay (ELISA)

Plasma from peripheral blood and placental tissue was collected to assess the fibronectin concentration using an enzyme-linked immunosorbent assay (ELISA) kit (R&D Systems, Minneapolis, MN, USA), following the manufacturer’s instructions. Briefly, each sample was applied to a microplate coated with capture antibody for 2 h at room temperature. A detection antibody conjugated to streptavidin–horseradish peroxidase was applied, followed by a color development solution (tetramethylbenzidine substrate) for 20 min. Color development was terminated by the addition of sulfuric acid, after which optical density was determined at 450 nm using a microplate reader (FlexStation 3, Molecular Devices, Sunnyvale, CA, USA). A fibronectin standard (R&D Systems, Minneapolis, MN, USA) was used as the control, and a standard curve was generated using a four-parameter logistic curve fit.

### 4.7. Western Blot Analysis

Lysates of treated cells or placental tissues were collected using immunoprecipitation (RIPA) lysis buffer (Millipore, Bedford, MA, USA). For signaling analyses, HTR-8/SVneo cells were treated with recombinant human fibronectin (5 μg/mL) or aspirin (1 mM) in serum-free media for different time intervals (0, 0.25, 0.5, 1, 2, 4, 8, and 24 h). After performing specific treatments, cells were harvested and collected for protein concentration analysis using a protein assay kit (Bio-Rad, Hercules, California, USA) according to the manufacturer’s instructions. Proteins were separated by sodium dodecyl sulfate-polyacrylamide gel electrophoresis (SDS-PAGE) and electrotransferred onto polyvinylidene fluoride (PVDF) membranes. After blocking in 5% bovine serum albumin (BSA), the membranes were incubated overnight at 4 °C with the following primary antibodies: Fibronectin (0.14 mg/mL, GeneTex, GTX112794, Hsinchu City, Taiwan), Vimentin (0.32 mg/mL, GeneTex, GTX100619, Hsinchu City, Taiwan), β-catenin (0.1 μg/mL, GeneTex, GTX101435, Hsinchu City, Taiwan), anti-phospho-p44/42 mitogen-activated protein kinase (MAPK) (Erk1/2) (Cell Signaling, #4370, Danvers, MA), anti-p44/42 MAPK (Erk1/2) (Abcam, Cambridge, UK), anti-phospho-Akt (S473) (Cell Signaling, #4060, Danvers, MA), anti-Akt (Cell Signaling, #9272, Danvers, MA), and β-actin (0.1 mg/mL, GeneTex, GTX100313, Irvine). The membranes were washed and incubated with goat anti-rabbit IgG antibody (HRP) (1:8000, GeneTex, GTX213110-1, Irvine, CA, USA) and goat anti-rabbit IgG antibody (HRP) (1:5000, GeneTex, GTX213111-1, Irvine, CA, USA). The results were then obtained using an enhanced chemiluminescence (ECL) commercial kit (EMD Millipore, Temecula, CA, USA) and a FluorChem™ R system (ProteinSimple, San Jose, CA, USA) and analyzed using ImageJ software (https://imagej.nih.gov/ij/download.html) (accessed on 15 July 2022).

### 4.8. Statistical Analysis

All values are expressed as the means ± SEM of at least three independent experiments. Statistical analyses were performed using GraphPad Prism version 6.0 (GraphPad Software Inc., San Diego, CA, USA). Differences between the control and treated groups (siRNA or recombinant human fibronectin) were evaluated using one-way ANOVA followed by Tukey’s post hoc test. *p* < 0.05 was considered statistically significant.

## 5. Conclusions

Our results demonstrate that placental FN was upregulated in patients with preeclampsia. Aspirin inhibited cellular FN expression and reverses FN-mediated trophoblast invasion and migration. This delineated the possible roles of FN in the pathogenesis of preeclampsia and the direct effects of aspirin on FN suppression, FN-regulated signaling, and cell motility in trophoblasts. Therefore, FN and the FN-mediated ERK and AKT signaling pathways could be potential diagnostic and therapeutic targets for preeclampsia prevention and intervention. By combining clinical data from preeclamptic patients and in vitro experiments, this study provides a plausible explanation for aspirin efficiency and its molecular mechanism of disease prevention.

## Data Availability

Data are contained within the article.
